# Sticky Platelet Syndrome: An Unrecognized Cause of Acute Thrombosis and Graft Loss

**DOI:** 10.1155/2018/3174897

**Published:** 2018-04-22

**Authors:** Fabio Solis-Jimenez, Hector Hinojosa-Heredia, Luis García-Covarrubias, Virgilia Soto-Abraham, Rafael Valdez-Ortiz

**Affiliations:** ^1^Internal Medicine Service, General Hospital of Mexico “Dr. Eduardo Liceaga”, Mexico City, Mexico; ^2^Transplant Service, General Hospital of Mexico “Dr. Eduardo Liceaga”, Mexico City, Mexico; ^3^Pathological Anatomy Service, General Hospital of Mexico “Dr. Eduardo Liceaga”, Mexico City, Mexico; ^4^Nephrology Service, General Hospital of Mexico “Dr. Eduardo Liceaga”, Mexico City, Mexico

## Abstract

**Introduction:**

Sticky platelet syndrome (SPS) is a prothrombotic disease that is not well recognized and difficult to diagnose.

**Case Report:**

We present a case of a 49-year-old diabetic woman on ambulatory peritoneal dialysis therapy who underwent a kidney transplant from living-related donor. The donor was her sister with whom she shared one haplotype and absence of donor specific antibodies. The posttransplant evolution was torpid, developing progressive deterioration, which made us suspect a failure in the graft. Doppler ultrasound reported renal vein thrombosis and hypoperfusion of the renal artery. Without clinical improvement, she required a reintervention that ended in graftectomy, in which the histopathological report showed negative C4d with medullary and cortical infarction. Hematological studies were negative for antibodies against phospholipids, with correct levels of proteins C and S and antithrombin. Platelet aggregometry studies were carried out, which were compatible with SPS.

**Conclusions:**

Recognition of SPS in pretransplant studies is difficult if there is no history of previous thrombotic events. However, we must consider this entity in cases of acute thrombosis and loss of the graft of uncertain origin.

## 1. Introduction 

Primary graft thrombosis occurs in 0.5% and 6% of renal transplants and usually results in graft loss [1]. Risk factors associated with the development of graft thrombosis have been identified, such as the use of peritoneal dialysis, retransplantation, prolonged cold ischemia (greater than 24 hours), and transsurgical hypotension [2–4]. However, in all patients presenting with thrombosis of the graft, an intentional search for possible primary thrombophilias should be carried out [4]. Retrospective studies performed in transplant patients with graft thrombosis have shown a higher incidence of protein C, protein S, factor V Leiden, and antithrombin deficiency [5]. However, in certain cases, the etiology of thrombosis remains uncertain and this is when a possible pattern of platelet hyperaggregation as sticky platelet syndrome (SPS) could be considered as the cause of thrombosis [6].

The SPS was first described in the 80s as a thrombophilia in which qualitative alterations of the platelet function increase its aggregation capacity, favoring in this way thrombosis with described cases of cerebrovascular disease, acute myocardial infarction, and ischemic retinopathy [7]. Below, we present the case of a patient without history of thrombosis who developed a sudden dysfunction of the graft after living kidney transplantation and whose final diagnosis was SPS.

## 2. Case Report

We report the case of a 49-year-old woman with O RhD positive blood group and a family history of premature death of her father due to cerebrovascular disease. In her surgical history, she underwent transsphenoidal surgery for pituitary adenoma at the age of 40, with replacement therapy with levothyroxine at a dose of 100 mcg per day. The patient was diagnosed with type 2 diabetes mellitus at the age of 29 and has been treated since then with long-acting insulin. She also has a diagnosis of CKD in treatment with automated peritoneal dialysis from 47 years of age. A living-donor kidney transplant protocol was initiated, related to her sister (healthy 39-year-old woman), with whom she shares one haplotype and specific negative anti-donor antibodies.

**Figure 1:**
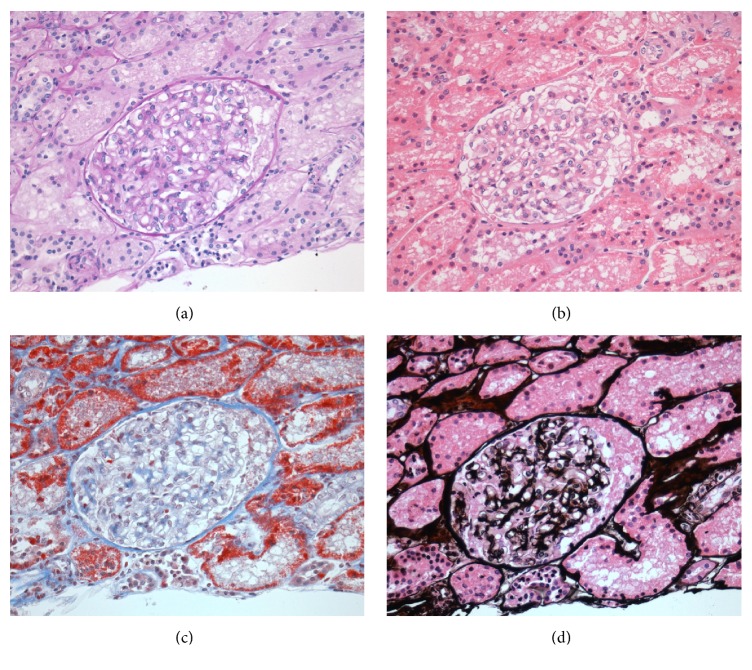
*Zero-time biopsy of the renal graft*. (a) Hematoxylin and eosin staining. (b) Periodic acid-Schiff staining. (c) Masson stain. (d) Silver methenamine stain. Renal tissue is visualized without significant vascular, tubular, and glomerular alterations. All photomicrographs are in 40x.

**Figure 2:**
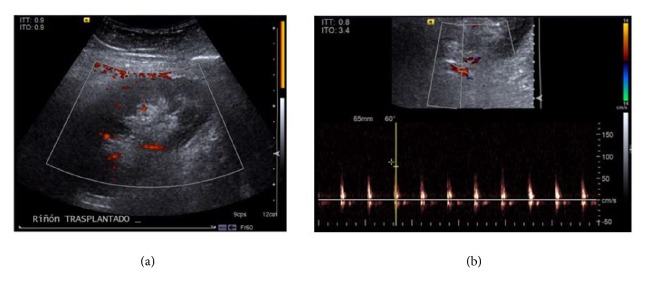
*Doppler ultrasound*. (a) Graft of the patient shows the absence of blood flow at the level of the renal cortex, arcuate arteries, interlobular arteries, and arterial anastomosis. (b) Duplex mode shows an arterial biphasic waveform with delayed acceleration, decreased systolic peak, and increased flow in diastole, which is a suggestive pattern of ischemia.

The induction scheme was performed with basiliximab and methylprednisolone, with a cold ischemia time of 120 minutes. During surgery, arterial anastomosis was observed with progressive decrease of the thrill. For this reason, it was necessary to dismantle the anastomosis followed by exploration, observation, and discharge of a thrombus from the renal artery. The anastomosis was completed with adequate graft perfusion. The renal biopsy at time zero was without significant vascular, glomerular, and interstitial tubule alterations (Figures 1(a)–1(d)). After surgery, the patient remained in oligoanuria. A Doppler ultrasound of the graft was performed, which showed the renal artery throughout its course with high resistance pulsatile flow, with an average systolic flow velocity of 58.7 cm/second and inversion of the diastolic flow. The segmental, interlobar, and arcuate arteries in the upper, lower, and middle poles showed decreased systolic flow with an average of 34.1 cm/second. Meanwhile, the renal vein could not be identified at the time of the study (Figures 2(a) and 2(b)).

**Figure 3:**
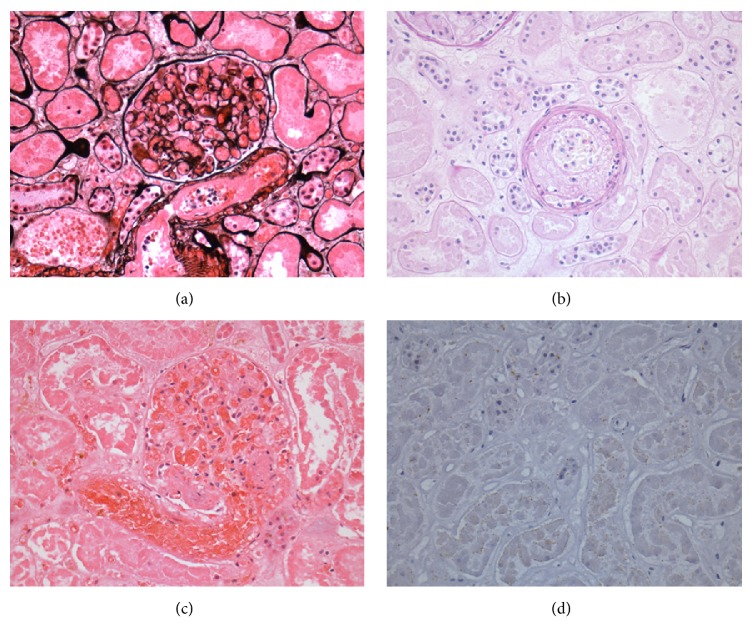
*Micrographs of graft nephrectomy (40x)*. Stained, respectively, with Jones' Methenamine (a), PAS (b), Hematoxylin and Eosin (c), and indirect peroxidase immunostaining for C4d (d). In all sections, fibrin thrombi adhered to the endothelium of the glomeruli ((a) and (c)) and the arteriolar walls (b) can be observed. C4d (d) was negative. All photomicrographs are in 40x.

**Figure 4:**
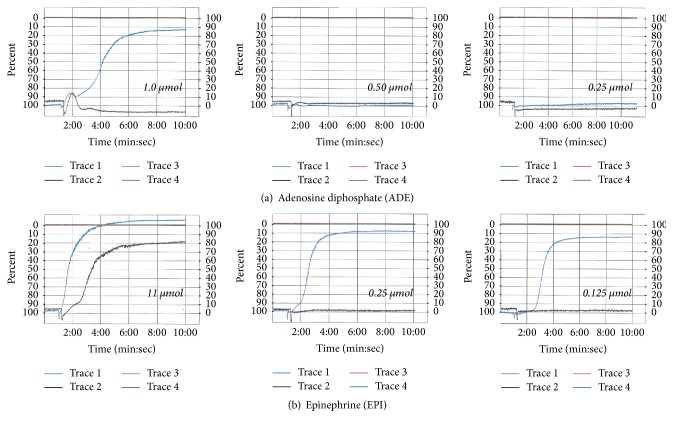
*Results of two platelet aggregometry tests: patient (blue line) and control (black line).* (a) From left to right, the percentage of platelet aggregation with decreasing doses of ADE is shown. At 1 *μ*mol, the patient presents platelet aggregation of almost 90% at 10 min. At lower concentrations of ADE, the pattern of aggregation is similar to that of the control (0.5 and 0.25 *μ*mol). (b) From left to right, the percentage of platelet aggregation with decreasing doses of EPI is shown. At 11 *μ*mol, the patient and control present platelet aggregation that reaches more than 80% at 10 min. In contrast to lower concentrations of EPI, while the control stops having platelet aggregation, the patient maintains a pattern of aggregation above 80% at concentrations of 0.25 and 0.125 *μ*mol.

Ultrasound diagnosis was a thrombosis of the renal vein with hypoperfusion of the renal artery. The patient was admitted to the operating room and as a macroscopic finding the renal graft showed a purplish coloration and pallor, absence of thrill, complete anastomosis, and the presence of an intrarenal venous clot. Due to the damage, graft transplantectomy was performed and the graft was sent for revision by a pathologist who reported diffuse medullary cortical infarction and acute thrombotic microangiopathy with negative immunohistochemistry for C4d (Figures 3(a)–3(d)). The postsurgical follow-up was performed with the search for potential thrombophilias. The quantification of protein C, protein S, and antithrombin was normal and the profile for anti-phospholipid antibodies was negative. In search of other pathologies, we requested studies of platelet aggregation, which showed platelet hyperaggregability at decreasing doses of epinephrine and normal aggregability associated with the exposure of adenosine diphosphate, compatible with sticky platelet syndrome type II (Figures 4(a) and 4(b)). The patient returned to automated peritoneal dialysis and management with acetylsalicylic acid as an antiplatelet agent was started.

## 3. Discussion 

Described for the first time in the Ninth Stroke and Cerebral Circulation Conference by Holiday et al. [8], the sticky platelet syndrome is defined as a qualitative alteration of the platelet function and of autosomal dominant inheritance, characterized by platelet hyperaggregation in vitro with low concentrations of adenosine diphosphate (ADE) and/or epinephrine (EPI) but with normal aggregation in response to collagen, arachidonic acid, ristocetin, and thrombin [9].

Depending on the pattern of platelet aggregation, three different types of sticky platelet syndrome have been described. In type I, hyperaggregation is evidenced with both ADE and EPI. Type II shows hyperaggregation only with EPI, and in type III, hyperaggregation is seen only with ADE [10]. There are no specific epidemiological data on SPS, because the studies have taken place in very exclusive population groups and in patients with thrombosis without apparent cause. Prevalence has been reported in patients with thrombosis without apparent cause between 17.6 and 28% [7, 11], while in women with miscarriages it has been reported in 20% [12] and in 41% of patients on hemodialysis with recurrent thrombosis of vascular access [13].

Within the clinical picture presented by these patients, arterial thrombosis is the most frequent manifestation of the disease, followed by venous thrombosis [14]. A study conducted by the National Center of Haemostasis and Thrombosis of Slovakia characterized 360 patients with SPS, describing 233 patients (64.7%) with arterial thrombosis and 127 (35.2%) with venous thrombosis [14]. Other characteristic data of SPS are the presentation in young adults with no apparent risk factors, in people with a family history of thrombosis, in women with repeat miscarriages, and in patients who have thrombosis in unusual sites (retinal circulation, cerebral sinuses, etc.) and episodes of thrombosis which occur even in spite of adequate anticoagulation [9].

One of the laboratory tests that help evaluate platelet function is aggregometry, which measures the ability of some substances to induce in vitro platelet activation and aggregation.

The diagnosis of SPS is made through a study of platelet aggregation in which platelet overaggregation is demonstrated when exposed to ADE and/or EPI. The most widely used method is turbidimetry, which measures the average platelet aggregation from the difference in optical density between platelet-rich plasma (PRP) and platelet-poor plasma (PPP), when an agonist is added as ADP, epinephrine, collagen, and ristocetin, to name a few; as the platelets are added, they allow a greater passage of light, decreasing the optical density, yielding the result through the aggregation curves as a function of time. Another method of measurement used is impedance, which measures the increase in resistance to the passage of electrical current through 2 electrodes by placing whole blood in contact with an agonist when platelets begin to aggregate [15].

In 2007, Mühlfeld et al. reported three cases in which patients had presented thrombotic complications after a kidney transplant and were diagnosed with SPS after posttransplant thrombotic episodes. Mühlfeld proposed that the increase in adrenaline secretion due to preoperative stress was likely to induce the typically abnormal aggregation pattern of SPS [6]. However, there is a possibility that other mechanisms may be involved, such as alloimmune vascular damage, postoperative hypertensive episodes, and the use of immunomodulatory drugs such as calcineurin inhibitors [6]. From the description of SPS, Mammen proposed that the underlying cause was in alterations of membrane glycoproteins and their role in platelet activation [16]. Although there have been multiple studies, to date, it has not been possible to find a genetic alteration that explains this syndrome. Different membrane protein polymorphisms have been studied, such as mutations in GPIIIaPlA A1/A2 and in Gas6 c. 834 + 7G>A, in which no statistically significant difference was found between the groups with SPS and controls [17, 18]. However, some polymorphisms of GP6 SNPs have been shown to be present more frequently in cases of SPS [19]. Despite these associations, there is still not enough evidence to define the etiology of SPS, which, due to its different forms of clinical presentation and inducibility characteristics of platelet aggregation, could have a multifactorial origin [9]

Because there are no treatment guidelines for SPS to date, the current recommendations are based on observations that acetylsalicylic acid (ASA) at low doses can normalize the pattern of platelet aggregation [14, 16]. For this reason, it is recommended to start treatment with ASA at doses of 80 to 100 mg every 24 hours [14]. However, for those patients who do not have an adequate response, it is recommended to scale the dose to 325 mg/day. The use of ADP inhibitors such as clopidogrel could be recommended only if, despite the escalation of the ASA dose, there is no normalization of the platelet aggregation pattern or there is any contraindication to the use of ASA [20]. Although monitoring of the efficacy of platelet antiaggregants is not standardized, it is recommended that once the treatment with ASA or clopidogrel has begun, the aggregation tests should be reevaluated in order to achieve adequate efficacy.

A targeted search for SPS should be recommended if there is a history of thrombosis without a specific cause or when the patient on the transplant and hemodialysis waiting list has reports of recurrent thrombosis of vascular access, particularly in renal transplant recipients.

The preoperative treatment of a transplant recipient when they have a proven SPS is controversial. Traditionally, it is described that there is an increased risk of bleeding during a surgical procedure (approximately 20% only with aspirin and up to 50% with aspirin and clopidogrel) [21]. However, there are studies in which it has been proven that the use of aspirin before and after transplantation reduces the risk of graft thrombosis without significantly increasing the risk of bleeding [22, 23]. These studies use low doses of aspirin (75–150 mg), which seems to be a strategy to be used in patients with SPS who will undergo kidney transplantation [24].

In conclusion, SPS is a poorly recognized entity but with an adverse prognosis in patients with kidney transplantation. Its recognition must be done in the pretransplant period to avoid adverse complications in the postoperative period. Its diagnosis requires evidence of platelet aggregation and its treatment is relatively effective with the use of antiplatelet agents.
